# Chronic recurrent multifocal osteomyelitis (CRMO) – advancing the diagnosis

**DOI:** 10.1186/s12969-016-0109-1

**Published:** 2016-08-30

**Authors:** M. R. Roderick, R. Shah, V. Rogers, A. Finn, A. V. Ramanan

**Affiliations:** 1 Department of Paediatric Immunology and Infectious Diseases, Bristol Royal Hospital for Children, Bristol, UK; 2 Department of Paediatric Rheumatology, Bristol Royal Hospital for Children, Upper Maudlin Street, Bristol, BS2 8BJ UK; 3 School of Clinical Sciences, University of Bristol, Bristol, UK

**Keywords:** Chronic recurrent non-bacterial osteomyelitis (CRMO), Pamidronate, Autoinflammatory, Cohort, Chronic recurrent multifocal osteomyelitis (CNO)

## Abstract

**Background:**

Chronic recurrent multifocal osteomyelitis (CRMO) is a little known inflammatory bone disease occurring primarily in children and adolescents. Delays in referral and diagnosis may lead to prolonged courses of antibiotics with in-patient care, unnecessary radiation exposure from multiple plain radiographs or bone scans and repeated surgery including bone biopsies.

Children (aged < 18 years) diagnosed with CRMO between January 2005 and December 2012, reviewed at Bristol Royal Hospital for Children were included and all available data collected. Information regarding CRMO was sent to all orthopaedic surgeons in the region in 2009.

The aim of the study was to examine the features of the cohort, to examine the length of time to diagnosis and to explore the criteria used for diagnosis with and without biopsy.

**Findings:**

Over an 8 year period, 41 patients were diagnosed with CRMO. Symptom onset occurred at a median of 9 years of age and time to diagnosis had a median of 15 months (range 0–92). Correlation coefficient analysis for time to diagnosis by year showed statistical significance with a decreasing trend. From the cohort data, diagnostic criteria were developed; applied retrospectively, 34 (83 %) children may have been diagnosed using the criteria, without a biopsy.

**Conclusions:**

The data suggest that increasing knowledge of this condition may shorten time to diagnosis. Use of the Bristol diagnostic criteria by an experienced clinician may obviate the need for biopsy in some patients.

## Background

Chronic recurrent multifocal osteomyelitis (CRMO), also known as chronic nonbacterial osteomyelitis (CNO), is an inflammatory bone disease occurring primarily in children and adolescents unfamiliar to many. It was first described in 1972 by Giedion as “an unusual form of multifocal bone lesions with subacute and chronic symmetrical osteomyelitis” [1].

There are now around 400 cases of CRMO described in the literature predominantly as case series. The true prevalence is difficult to assess as it is a little-known disease and likely to be vastly underdiagnosed [2].

The consistent feature of CRMO is the insidious onset of pain with swelling and tenderness localised over the affected bones. Involvement of the clavicle is the classical picture; however, the metaphyses and epiphyses of the femur, tibia or humerus are also frequently affected. Lesions may occur in any bone, including vertebrae [3, 4].

CRMO has recently been classified as an autoinflammatory disorder (rather than autoimmune). These inflammatory conditions are characterised by episodes of systemic inflammation including serological signs of inflammation (CRP, ESR, IL-6, TNF-α) occurring in the absence of autoantibodies, pathogens or antigen-specific T cells [5, 6].

As the condition is obscure to many doctors, and commonly being misdiagnosed as bacterial osteomyelitis, delays in referral and correct diagnosis may lead to prolonged courses of antibiotics (often given intravenously in hospital), unnecessary radiation exposure from multiple plain radiographs or bone scans and repeated bone biopsies before a diagnosis is made.

The diagnosis of CRMO is made by exclusion of other diseases, and commonly requires a bone biopsy in order to exclude infection, neoplasia or langerhans’ cell histiocytosis.

## Findings

The Bristol Royal Hospital for Children provides a local and a tertiary referral service for specialist paediatric rheumatology care for families across the South West of England and nationally.

Children (aged less than 18 years) who had been previously diagnosed with CRMO between January 2005 and December 2012, reviewed at Bristol Royal Hospital for Children were included in the review. These diagnoses had been made following a variety of presentations, using clinical, radiological and sometimes histological findings.

In 2009, the Bristol rheumatology and immunology department wrote to all the orthopaedic surgeons in the South West of England. This manuscript aimed to raise awareness of CRMO as a condition and recommended referral to the Bristol service.

We retrospectively reviewed clinical, biological and radiological data on children diagnosed with CRMO during the specified period. Complete medical history including age at first symptoms, age at diagnosis, sex, constitutional symptoms at disease onset, number of painful sites involved, family history, past history and treatment history were recorded from the clinical notes. The notes were available for review on all 41 patients, all 37 magnetic resonance images (MRI) scan reports were reviewed but only 36/41 (88 %) plain radiograph results were available.

Inflammatory markers and bone biopsy results were recorded and imaging reports of plain radiography, bone scans and whole body – magnetic resonance imaging (WB-MRI) were collected.

A descriptive analysis of patient characteristics was performed obtaining data from peripheral hospitals where necessary. Data was entered into a table and was examined by two independent researchers. The length of time (in months) from the onset of symptoms to diagnosis for each child was plotted on a scatter chart and the correlation coefficient was calculated with *p* values using MS Excel.

Forty one patients (31 female and 10 male) were diagnosed as CRMO and assessed at the Bristol centre over an 8 year period to December 2012. The ethnicity was not recorded in the notes; patients were referred from the South West of England and Wales.

The median age at the onset of first symptom was 9 years (range 1–13) and the median age at diagnosis was 11 years (range 1–17). The median time from onset of symptoms to diagnosis was 15 months (range 1–92). Bony pain with or without swelling was the most common presenting complaint. Patients were most commonly initially referred to an orthopaedic surgeon (21 patients, 51 %), or a general paediatrician (10 patients, 24 %). One patient was referred to a general surgeon before being referred to the Bristol centre.

Before the diagnosis of CRMO was made, the most common differential diagnoses were infective osteomyelitis, malignancy e.g. Ewing’s sarcoma, Langerhans’ cell histiocytosis, non-specific musculoskeletal disorders, juvenile idiopathic arthritis and viral infections.

The initial clinical presentation was in a single site in 22 patients (54 %) but after each patient had been fully evaluated by different methods (clinical examination, plain x-ray, MRI or bone scan) only 10 patients (24 %) had single site involvement (6 of which were clavicle alone).

The most common bones involved at presentation (by clinical assessment only) were tibia (27 patients, 66 %) and clavicle (14 patients, 34 %); spinal symptoms were present in six patients (15 %). Only five (12 %) patients had symmetrical symptoms on presentation. Constitutional symptoms (e.g. fever, malaise), skin lesions (pustules or psoriasis) and joint swelling were observed in 6 (15 %), 4 (10 %) and 7 (17 %) patients respectively. No associated clinically apparent inflammatory bowel disease was seen in our cohort.

In all patients complete blood count, renal function and liver function tests were normal. A C-reactive protein (CRP) level was available in 28 patients (median value 7 mg/l, range 1–30 mg/l) and mildly raised in 14 (>10 g/L). Erythrocyte sedimentation rate (ESR) was measured in 19 patients (median value 27 mm/h, range 1–72) in 16 of whom it was raised (≥11 mm/h).

**Fig. 1:**
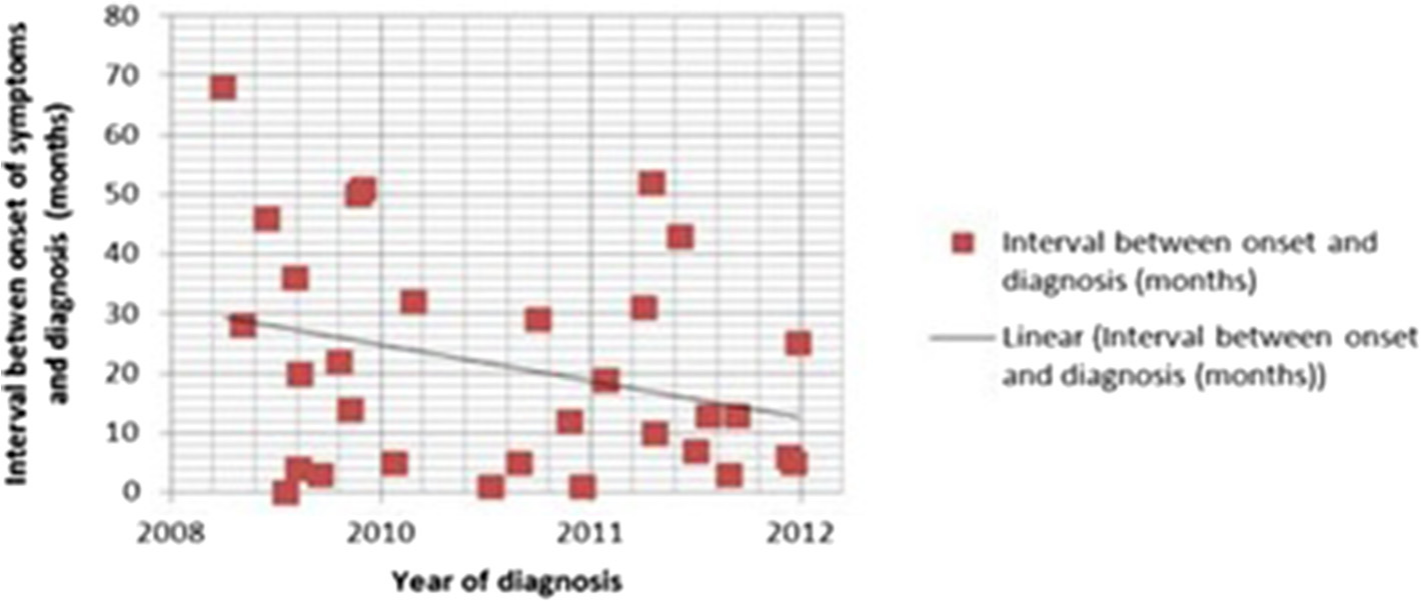
Correlation plots between interval between onset of symptoms and year of diagnosis for all 41 patients. *R* = −0.29

The length of time (in months) from the onset of symptoms to diagnosis for each child was plotted on a scatter chart showing the trend line for all children referred (Fig. 1). The correlation coefficient *R* = −0.29 (*P* = 0.03 using a directional test) showed a statistically significant decreasing trend for time to diagnosis. Bone biopsy had been performed in a total of 32 patients (78 %), 29 of these were biopsied before being referred to our centre; 20 of these patients had one biopsy, 11 patients had undergone biopsy on 2 occasions and 1 patient had a biopsy on three occasions before the diagnosis was made.

In 24 patients the pathology report described inflammation on biopsy specimens while seven patients had no obvious inflammatory changes. One patient’s biopsy was reported as showing features consistent with a chondroma (however, subsequently, WB-MRI showed multiple typical CRMO lesions). Plasma cell infiltrates were seen in 16 patients (50 %). Reactive bone changes, predominantly fibrosis, were seen in 23 out of 32 patients (72 %); only two of these had no evidence of inflammation. All specimens were sent for culture and all but one were negative. One biopsy specimen grew *S. aureus* on enrichment culture but there were no histological features of infection or inflammation; an MRI showed symmetrical lesions and repeat biopsy and culture (off antibiotics) was negative.

Twelve children were referred without biopsy of whom nine did not require a biopsy for diagnosis. Five of these had typical multiple lesions on MRI scan, one had a single affected area and had a sibling with typical CRMO, and three patients had a solitary lesion of the clavicle.

Plain radiograph of the major symptomatic site was available in 36 out of 41 patients. It was abnormal in 28/36 (78 %) revealing lytic lesions in 18 (50 %), areas of sclerotic bone in 19 (53 %) and periosteal reaction in 12 patients (33 %). Among the eight patients with normal plain radiographs, the median length of symptoms was 3.5 months compared with 8.5 months in the abnormal group (not significant, *p* >0.05 by Mann–Whitney U test). Whole body MRI detected an abnormal lesion at the symptomatic site in seven of these eight patients with normal x-ray. Fourteen patients had had a bone scan prior to being seen by the rheumatology team. In three patients (3/14) the bone scan identified an asymptomatic site.

Whole body MRI has been described as being helpful in reaching the diagnosis [7, 8]. Out of the 41 patients, 37 (90 %) patients were evaluated with MRI, 30 with whole body MRI and 7 with localised imaging. In total 162 lesions were detected (see Table 1). Of the 162 lesions identified by imaging, 47 (29 %) were asymptomatic and detected only by MRI. Unifocal involvement was observed in the clavicle (6 patients), mandible (1 patient), tibia (1 patient) and femur (1 patient). 32 spinal lesions were detected in 13 patients, 6 of these had only one spinal lesion with other lesions elsewhere. Two patients only had spinal involvement but both of these had more than one lesion, and neither was evaluated by whole body MRI (therefore silent lesions may have been present but undetected).

**Table 1:** Distribution of different lesions by imaging

Site affected	Number of detected lesion	% of total lesions *n* = 162
Pelvis	14	9
Femur	16	10
Tibia	40	25
Fibula	8	5
Small bone in foot	16	10
Humerus	7	4
Radius	5	3
Ulna	4	2
Mandible	1	1
Clavicle	12	7
Ribs	6	3
Sternum	1	1
Vertebra	32	20
*- Cervical*	*(2)*	*(1)*
*- Thoracic*	*(18)*	*(11)*
*- Lumbar*	*(3)*	*(2)*
*- Sacral*	*(8)*	*(5)*
*- Coccyx*	*(1)*	*(1)*
Total	162	100

Twenty two patients (54 %) received at least one course of intravenous antibiotics before the diagnosis was made. Fourteen (34 %) patients did not require therapy other than non-steroidal anti-inflammatory drugs (NSAIDs). Three patients were treated with corticosteroids but none of them reported associated improvement. Six patients (with pain resistant to treatment with NSAIDs) were treated with methotrexate; two patients made no response, four patients went into remission but two of them relapsed on follow up. One patient was treated with sulfasalazine and responded well, remaining clinically asymptomatic after 4 years on treatment; however, repeat MRI showed four new lesions in this patient.

Where there was no response to NSAIDs, and other agents (where given), pamidronate (as intravenous cycles of 1 mg/kg/day for 3 consecutive days) was used to seek to control disease activity. By the end of 2012, twenty-two children (54 %) had had some treatment with pamidronate. Thirteen had completed at least one year of treatment with 11 children having MRI both before and after treatment; the two children without MRI scans were treated before 2010 and after treatment both reported a good response and were discharged from follow-up. Seven children had started pamidronate therapy but had not yet completed treatment at the time of writing. Two patients had started treatment but had given up after nine months as their symptoms were not improving. Of these two children (who did not complete treatment), one had improved on follow-up and the other was commenced on biological therapy with etanercept in 2012. These findings are published in more detail elsewhere [9].

Of the thirteen patients who had completed one year of pamidronate at analysis, nine (69 %) patients had substantial relief from pain (no longer requiring any analgesia) after starting pamidronate therapy, and four had ongoing significant pain. Two of these (with ongoing pain) had new lesions identified on MRI and the other two had persistent disease activity on MRI albeit improved from their initial scans.

As expected with bisphosphonate therapy, flu-like symptoms with fever and myalgia were very common particularly during the first cycle although, with prior warning, all patients tolerated therapy very well. No major side effects were observed.

## Conclusions

Our findings suggest that raising the awareness of CRMO may lead to earlier diagnosis. The protracted interval of 15 months (median) between symptom onset and diagnosis is well recognised and is partially explained by the lack of specific clinical, laboratory and imaging findings, and the difficulty in distinguishing the inflammation from infection. In spite of being described in 1972 [1], CRMO is still not well recognised and is likely to be substantially more common than is currently diagnosed. Delay in diagnosis can lead to prolonged admissions for intravenous antibiotics (sometimes multiple admissions), and multiple biopsies which, it can be argued, are rarely necessary for the diagnosis of CRMO.

It is likely that in places where CRMO is seen less commonly, the diagnosis will be forgotten if publications or specific awareness-raising methods are not utilised.

Multifocal involvement is common but may be subclinical; distinguishing between CRMO and bacterial osteomyelitis is made easier when multiple sites are evident clinically or on imaging. Using WB-MRI scans on all children with suspected unifocal osteomyelitis is unlikely to be practical or necessary; however, once CRMO has been considered WB-MRI along with consideration of other typical features may make the diagnosis more obvious, and potentially avoid the need for biopsy.

**Fig. 2:**
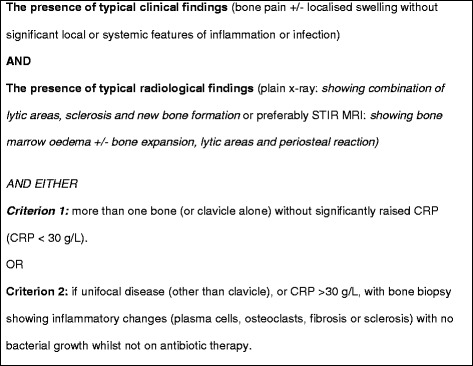
Bristol diagnostic criteria for CRMO

On the basis of the findings of this cohort, the criteria in Fig. 2 were formulated by the authors of the paper for use by the department. Using these criteria retrospectively, thirty-four children could have been diagnosed by criterion 1, with 6 children requiring a biopsy (criterion 2) for diagnosis, either for a solitary lesion (not clavicle) or because of atypical features such as very young age (<2 years). One child did not fit these criteria as she had only ankle involvement but her symptoms were mild, her sibling had a diagnosis of CRMO, and symptoms responded well to NSAIDs (making other causes of bone inflammation unlikely). Some patients with CRMO may have a markedly raised CRP but these are likely to require a biopsy in order to exclude an infectious agent.

Twelve children were without biopsy at referral of whom nine did not require a biopsy for diagnosis. Using these criteria for diagnosis provides a framework for avoiding biopsy in children with typical disease; however, these criteria cannot be validated by this retrospective audit as the clinicians and/or radiologist may have been aware of a biopsy report when making the diagnosis. It is very straightforward to declare the MRI findings as ‘typical’ for CRMO whilst holding a bone marrow report with suggestive features. The clinical vignette and available imaging would need to be shown to a blinded audience in order to validate these criteria objectively.

## Abbreviations

CNO: Chronic nonbacterial osteomyelitis
CRMO: Chronic recurrent multifocal osteomyelitis
CRP: C-reactive protein
ESR: Erythrocyte sedimentation rate
MRI: Magnetic resonance image
NSAID: Non-steroidal anti-inflammatory drug
WB-MRI: Whole body magnetic resonance image
